# Transcytosis of *Bacillus subtilis* extracellular vesicles through an *in vitro* intestinal epithelial cell model

**DOI:** 10.1038/s41598-020-60077-4

**Published:** 2020-02-20

**Authors:** Ana Paula Domínguez Rubio, Jimena Martínez, Marcos Palavecino, Federico Fuentes, Christian Miquel Sánchez López, Antonio Marcilla, Oscar Edgardo Pérez, Mariana Piuri

**Affiliations:** 1 Departamento de Química Biológica, Facultad de Ciencias Exactas y Naturales, Universidad de Buenos Aires, Buenos Aires, Argentina; 2 Instituto de Química Biológica de la Facultad de Ciencias Exactas y Naturales, Universidad de Buenos Aires, Consejo Nacional de Investigaciones Científicas y Técnicas, Buenos Aires, Argentina; 3 Instituto de Medicina Experimental, Consejo Nacional de Investigaciones Científicas y Técnicas, Buenos Aires, Argentina; 4 Àrea de Parasitologia, Departament de Farmàcia i TecnologiaFarmacèutica i Parasitologia, Universitat de València, Burjassot, Valencia, Spain; 5 Joint Research Unit on Endocrinology, Nutrition and Clinical Dietetics, Health Research Institute La Fe, Universitat de Valencia, Valencia, Spain

**Keywords:** Cellular imaging, Cellular microbiology

## Abstract

Bacterial EVs have been related to inter-kingdom communication between probiotic/pathogenic bacteria and their hosts. Our aim was to investigate the transcytosis process of *B. subtilis* EVs using an *in vitro* intestinal epithelial cell model. In this study, using Confocal Laser Scanning Microscopy, we report that uptake and internalization of CFSE-labeled *B. subtilis* EVs (115 nm ± 27 nm) by Caco-2 cells are time-dependent. To study the transcytosis process we used a transwell system and EVs were quantified in the lower chamber by Fluorescence and Nanoparticle Tracking Analysis measurements. Intact EVs are transported across a polarized cell monolayer at 60–120 min and increased after 240 min with an estimated average uptake efficiency of 30% and this process is dose-dependent. EVs movement into intestinal epithelial cells was mainly through Z axis and scarcely on X and Y axis. This work demonstrates that EVs could be transported across the gastrointestinal epithelium. We speculate this mechanism could be the first step allowing EVs to reach the bloodstream for further delivery up to extraintestinal tissues and organs. The expression and further encapsulation of bioactive molecules into natural nanoparticles produced by probiotic bacteria could have practical implications in food, nutraceuticals and clinical therapies.

## Introduction

Functional foods benefit human health beyond their basic nutritional properties^1^. These types of food contain chemical or biological compounds that provide health benefits with the concomitant reduction of disease risk. Functional food and drinks are products enriched with nutrients or substances such as probiotics, prebiotics or omega 3 fatty acids^2^. Probiotics are live microorganisms, which when administered in adequate amounts confer a health benefit to the host^3^.

How food products modulate host physiology remain mostly unknown^4^. An interesting approach for the prevention and treatment of various diseases is by dietary interventions, which alter the gut microbiota. Gut microbiota can impact on different systems, i.e. digestive, immune and neuroendocrine. The link between changes in the gut microbiota to gastrointestinal and extraintestinal disorders has recently begun to emerge^5,6^. In this line, probiotics act through a wide repertoire of mechanisms that positively affect composition and/or function of the gut microbiota^7^. However, the way that probiotics exert their benefic effect must still be elucidated^8^.

There have been reports about the use of *Bacillus spp*. as probiotics for 50 years and one of the species that has been studied is *Bacillus subtilis*^9,10^. Endospore formers, such as *B. subtilis*, are interesting because they produce heat-stable spores. This could be a great advantage for the formulation of probiotic products that can be stored in a desiccated form without deleterious effects on bacterial viability^10^. *Bacillus subtilis* spores can germinate in the GIT and this differentiation occurs shortly in comparison to laboratory conditions^11^. *B*. *subtilis* is the dominant functional bacterium in all naturally fermented soy-based foods in Asian countries^12^ and cause a transient alteration of human intestinal microbiota^13,14^. In the case of pathogens belonging to this genera, such as *B. anthracis* and *B. cereus*, the entry into the GIT is an essential part of their virulent life cycle^15,16^.

The gastrointestinal mucosal surface is the primary interface between probiotics living in the intestine and host organs and systems^17^. The small intestine consists of heterogeneous cell populations, and absorptive enterocytes represent approximately 90% of the cells. *In vitro* models have been used to predict human intestinal absorption^18–20^. In this line, Caco-2 human cell line has been extensively used as a model for intestinal barrier studies, as a result of their physiological and morphological differentiation to enterocytes^19^. Differentiated Caco-2 cell model shows a good correlation between transport and bioavailability for lipid-based nanoparticles^21–27^.

The communication between mammalian gastrointestinal tract (GIT) and probiotics could in part occur through bacterial EVs and other mediators^28,29^. These led to the hypothesis that EVs could cross the epithelium of gastrointestinal mucosa and reach the bloodstream to be delivered to extraintestinal tissues and organs^30^.

Extracellular vesicles (EVs) seem to be an universal way of communication between cells as its production was reported for archaea, bacteria and eukarya^31^. These nanostructures contain biomolecules as proteins, lipids, nucleic acids and bioactive compounds. Even more, EVs can be applied in different fields as biomedicine and nanotechnology^32^. EVs are considered natural nanoparticles with potential application as delivery systems^33,34^. Several reports describing loading cargos in eukaryotic EVs such as siRNA^35^, mRNA^36^, proteins^37^ and chemotherapeutics^38^ have shown that EVs can function as nanocarriers. From a nanostructural point of view, EVs are membrane vesicle units constituted by lipids and surface proteins that offer advantages for cellular uptake over existing lipid-based delivery systems^39,40^. Advantages of nanocarriers include an increased solubility, higher colloidal stability and circulation time in the body as well. Additionally, overcoming the natural barriers of the body results crucial for oral delivery and to reach the blood for distribution to organs and tissues^41^.

EVs have been reported in Gram-positive bacteria, with a size between 10 and 400 nm, and have been related to bacterial physiology and its probiotic and pathogenic effects^42^. The diversity in cargo molecules contained in EVs might explain their variety of described roles ranging from bacterial competition, genetic material exchange, survival, host immune modulation and evasion, as well as infection^43^. As examples, *Lactobacilli* EVs are able to dampen pro-inflammatory cytokine response^44^, *Bifidobacterium longum* EVs effectively alleviated food allergy response in a mouse model^45^, and *Lactobacillus* EVs enhance host immune responses against vancomycin-resistant enterococci^42^.

The isolation and characterization of EVs from *B. subtilis* was reported for the first time by Brown *et al*. (2014), and several authors have further deepened these studies^46–49^. EVs formation occurs over the entire cellular lifespan of these spore forming bacteria and this probably does not escape what happens in GIT^14^. In this context, it is important to notice that the proteome of EVs formed during sporulation or vegetative growth differs^49^.

It has been previously demonstrated the internalization of Group B *Streptococcus* EVs by HeLa cells and from other Gram-positive bacteria^43,50^. But so far, no information is available on the internalization of intact *Bacillus spp*. EVs or their transport across human polarized epithelial cell monolayer.

The aim of this work was to explore the transcytosis process of EVs derived from the probiotic strain *B. subtilis*. To this end we used an *in vitro* transwell model of intestinal epithelial cell barrier. The transport of fluorescent-labeled EVs was visualized by Confocal Laser Scanning Microscopy (CLSM), and in combination with Confocal Time Lapse Imaging, we were able to determine the EVs trajectory within the Caco-2 cells. Transcytosis of EVs was also estimated using fluorescent measurements and Nanoparticle Tracking Analysis (NTA).

## Results and Discussion

### EVs uptake and internalization in Caco-2 cells determined by confocal Laser scanning microscopy analysis

**Figure 1:**
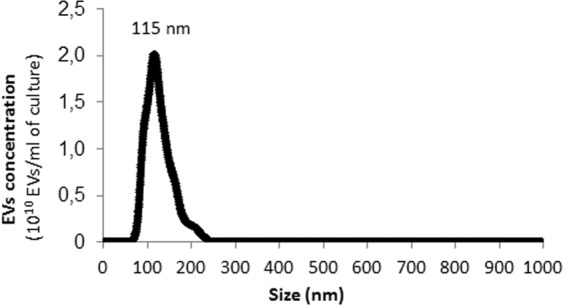
Representative plot of EVs concentration isolated from *B. subtilis* 168 versus particle size obtained by nanoparticle tracking analysis (NTA). Mean ± SD, n = 3.

We utilized our established protocol for the specific isolation of *B. subtilis* 168 EVs from stationary phase cultures^51^. EVs were quantified by NTA and revealed a unique population of 115 ± 27 nm (Fig. 1). The peak obtained ranged from 71 nm to 230 nm (n = 3). The EVs size obtained from NTA data was consistent with measurements previously obtained from Dynamic Light Scattering (DLS)^46,51^.

**Figure 2:**
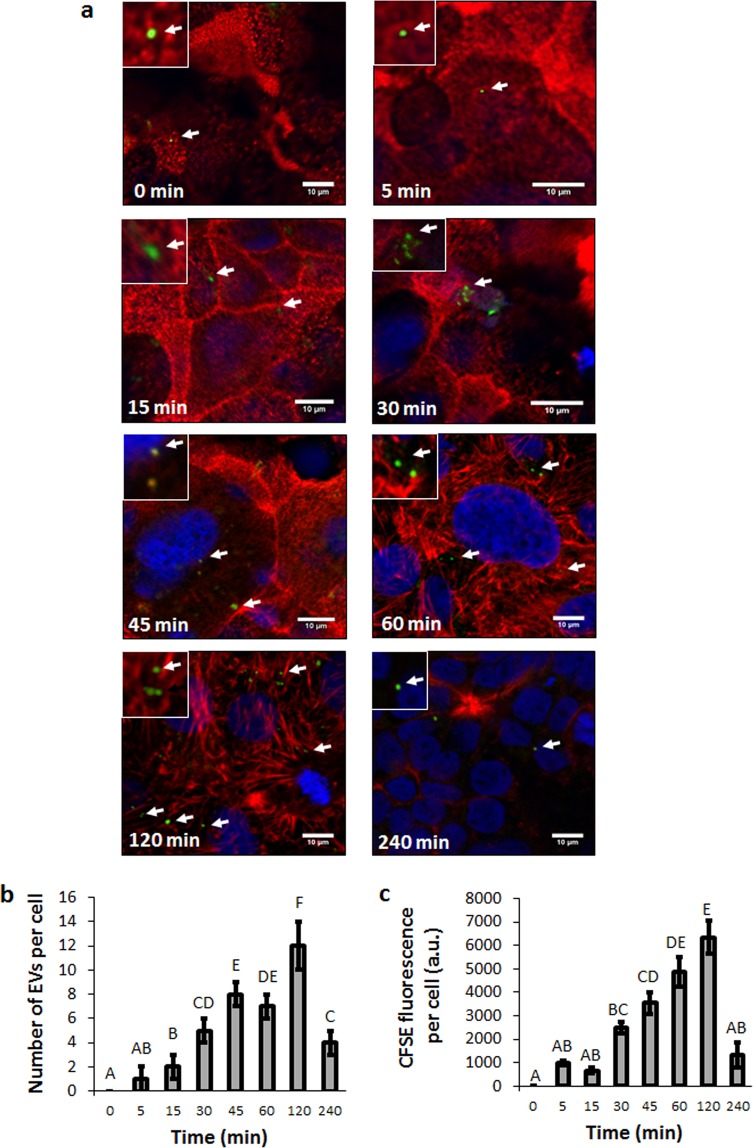
*B. subtilis* EVs are taken up efficiently by Caco-2 cells in a time-dependent active manner. Caco-2 cells differentiated monolayer were incubated with CFSE-labeled EVs for different times (0, 15, 30, 45, 60, 120 and 240 min). After incubation, cells nuclear DNA was stained with To-Pro3 and actin filaments were stained with Rhodamine Phalloidin, and images visualized by CLSM. (**a**) Arrows point out EVs. (**b**) Number of EVs internalized per cell at the different times of incubation. (**c**) CFSE fluorescence per cell at the different times of incubation. In b and c data represents Mean ± SD. One-way ANOVA with Tukey’s post hoc test were carried out (n = 3). Means with a common letter are not significantly different (p > 0.05).

The formation of a monolayer of Caco-2 cells was confirmed by the analysis of CLSM images. These cells presented a polarized morphology, with the apical face, rich in F-actin, constituting the brush border (red), and the basal region containing the aligned cell nuclei (blue) (Supplementary Fig. S1 and Fig. 2a).

To study the uptake and internalization of EVs by Caco-2 cell monoloayers, approximately 1.3 × 10^9^ CFSE-labeled (5-(6) carboxyfluoresceindiacetate N-succinimidyl ester) EVs from *B. subtilis* 168 were added to Caco-2 cells culture. At different time points cells were fixed, stained and subjected to CLSM (Fig. 2a and Supplementary Fig. S2). At the beginning of the incubation, the majority of CFSE-EVs (green) were found on the brush border of the enterocyte-like cells. At longer incubation time points most of the fluorescent signal measured was intracellular, suggesting active uptake and internalization of intact EVs (Fig. 2a). CFSE labels EVs protein cargo as well as transmembrane proteins *via* the action of intravesicular esterases, allowing only the detection of intact EVs^52,53^. The amount of EVs incorporated into Caco-2 cells seemed to be time-dependent. The maximum EVs uptake by cells occurred at 120 min, and decreased at 240 min (Fig. 2a,b). At 240 min, most of the CFSE-EVs observed were located on the basal face (Fig. 2a and Supplementary Fig. S2). As expected, CFSE fluorescence levels inside Caco-2 had a similar pattern to that obtained from the quantification of EVs per cell (Fig. 2b,c). It should be noted that the measured areas of the fluorescent EVs could not reflect their actual size because the point-spread function contributes to some degree of spreading (*i.e*., blurring)^54^. In this case, the theoretical lateral resolution for the fluorescent microscope is 183 nm (diameter=λem/2 N.A.; λem = 521 nm and N.A. = 1.42)^51^. The reduction of signal inside the cells, at 240 min in comparison to 120 min (n = 3, p < 0.05), could be explained by the release of the EVs and/or degradation of the CFSE dye in lysosomes (Fig. 2b,c). For that reason, we decided to carry out the transcytosis assays with a maximum incubation period of 240 min.

Our results are in accordance with Li *et al*. (2017), which described that *Lactobacillus plantarum* EVs could be internalized into Caco-2 cells^42^. Further research would be necessary to elucidate whether this is a cell type-specific process. Results from other studies showed that fluorescently labeled EVs can be taken up by virtually all cell types tested, whereas others suggest that vesicular uptake is a highly specific process which can only occur when cells and EVs share the right combination of ligand and receptor^55^. For example, *ex vivo* bone marrow–derived mast cells have a higher uptake ability compared to other cell types, and can efficiently take up EVs from *Enterococcus faecalis* as well as from *Bifidobacterium longum*, during an incubation period of 120 min^45^. Taking into account these data, Caco-2 cells would be capable of internalizing EVs from different probiotics strains.

**Figure 3:**
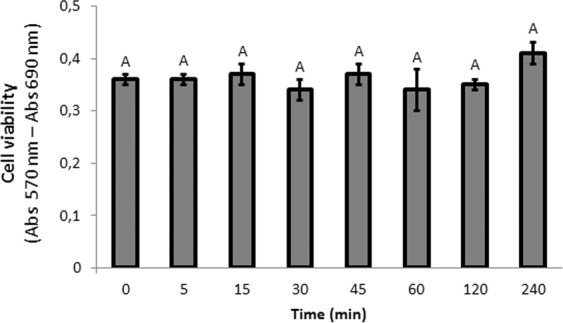
EVs derived from B. subtilis 168 did not affect cellular proliferation, viability or cytotoxicity of Caco-2 cells. Data were obtained from MTT Assay. Mean ± SD. One-way ANOVA (n = 8). Means with a common letter are not significantly different (p > 0.05).

Importantly, we tested that EVs from *B. subtilis* 168 collected from stationary phase cultures had no effect on Caco-2 monolayer cellular proliferation, viability or cytotoxicity at the evaluated incubation time points (n = 8, P > 0.05) (Fig. 3). In line with this observation, EVs from the probiotic strain *L. plantarum* do not cause any significant toxicity to Caco-2 cells after 24 h incubation^42^. However, EVs from *Lactobacillus rhamnosus* were shown to have a significant cytotoxic effect on a human liver epithelial cell line (HepG2) when incubated at an intermediate concentration for 24 h^56^. Concerning to the pathogenic bacteria *Staphylococcus aureus*, EVs of different strains show different cytotoxic activity toward host cells. The presence or absence of cytotoxicity may result from proteome differences in *S. aureus*-secreted EVs, but further analysis would be necessary to identify cytotoxic factors in these EVs^57,58^.

### Bacterial EVs transcytosis across Caco-2 cells as determined by fluorescence and nanoparticle tracking analysis measurements

In general terms, EVs transport across cellular barriers could be achieved either by a paracellular route or a transcellular route. The paracellular route corresponds to the passage between epithelial cells *via* transient disruption of the tight junctions that interlock adjacent cells. Instead, the transcellular route corresponds to the internalization, endocytosis, transport across the cell body and secretion at the opposite cell surface (transcytosis). TEER is an accepted technique to measure quantitatively the integrity of epithelial cells monolayer, which is determined by the tight junction dynamics. This approach is used to evaluate drugs or lipid-based nanoparticles transport^25,59^.

**Figure 4:**
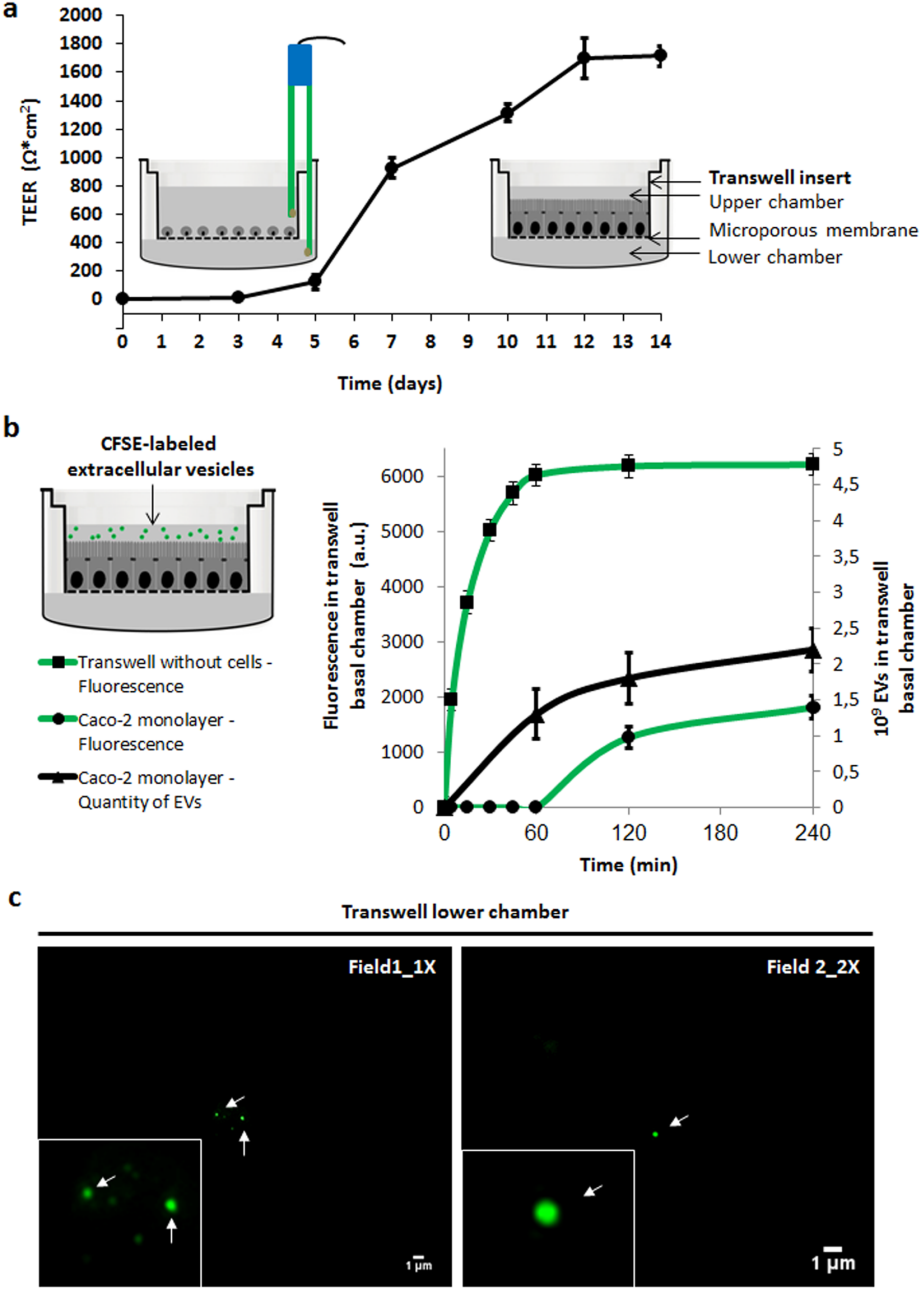
Intact *B. subtilis* 168 EVs are transported across Caco-2 cells in a time-dependent active manner. (**a**) TEER was measured every 2–3 days to follow the Caco-2 cells monolayer formation. On day 14 after cell seeding, TEER measurement stabilized and Caco-2 cells monolayers were used for transcytosis assays. (**b**) CFSE-labeled EVs were incubated with Caco-2 cells in the upper chamber of transwell system. CFSE fluorescence and the number of EVs (NTA) were measured of the medium collected from the lower chamber at different times. Mean ± SD (n = 3). (**c**) After 120 min incubation CFSE-labeled EVs were visualized using Epi-fluorescence Microscopy. Arrows point out EVs.

In our assays, Caco-2 cells were differentiated on transwells and TEER was measured every 2–3 days. On day 14 after cell seeding, TEER measurement stabilized at 1714 Ω*cm^2^ ± 72 Ω*cm^2^, and Caco-2 cells monolayers were then used for transcytosis assays (Fig. 4a). Validation of Caco-2 permeability barrier in this model was also done using immunostaining of tight junctions with anti-occludin primary antibodies and Alexa 488 labeled secondary antibodies on days 3 and 14, showing no disruption of the barrier (Supplementary Fig. S3).

To carry out the transcytosis assays, CFSE-labeled EVs were incubated with Caco-2 cells monolayers in the upper chamber. After different time points, fluorescence was measured in the medium collected from the lower chamber. Fluorescence was first detected at 120 min and the intensity of the signal increased 43% after 240 min of incubation (Fig. 4b). Transwells without cells were used as a control of passive diffusion. In this case, just after 5 min of incubation fluorescence levels were detected in the medium collected from the lower chamber and increased until 60 min, remaining stable for the rest of the incubation time.

To estimate the uptake efficiency of EVs, we compared the fluorescence units recovered in the lower chamber to the fluorescence from CFSE-labeled EVs seeded on the upper chamber (see Mat & Met). Considering that 6212 fluorescence arbitrary units (a.u.) were seeded (approximately 5 × 10^9^ EVs), EVs uptake was 20% ± 2% at 120 min and 29% ± 13% at 240 min. At both time points, the fluorescence units remaining in the upper chamber plus the fluorescence units recovered in the lower chamber equaled 95% of the total fluorescence seeded in the transwell. We speculate that the remaining 5% of the total fluorescence might be inside the cells within the compartments of endocytosis and/or transcytosis pathways and/or degradation path through lysosomes^60,61^.

In order to quantify the number of EVs transcytosed through Caco-2 monolayers we analyzed the samples by NTA. As mentioned before, about 5 × 10^9^ EVs were seeded in the upper chamber. EVs were first detected in the lower chamber after 60 min of incubation (Fig. 4b). The number of EVs measured in the lower chamber increased 38% at 120 min and up to 76% at 240 min. Based on the measurement of fluorescence, the appearance of EVs only after 60 min of incubation was unexpected but might be explained by a higher sensitivity of NTA compared to fluorescence to detect the presence of nanoparticles in the sample^62^.

We further estimated the efficiency of uptake using the values obtained by NTA. In this case, 27% ± 11% of the seeded EVs were obtained in the lower chamber at 60 min, 38% ± 14% at 120 min and 46% ± 14% at 240 min. Even though for quantification of EVs by NTA we selected those particles in a range from 71 nm to 230 nm, we could not discard the presence of EVs produced by Caco-2 cells (n = 3).

In order to corroborate that the measured particles corresponded to the transcytosed *B. subtilis* EVs, we performed a dose-dependent curve using an incubation period of 240 min (Supplementary Fig. S4). As seen in Supplementary Fig. S4 the number of recovered EVs in the lower chamber increased concordantly with and increment of the seeded EVs. An efficiency of uptake of 19% ± 5% was observed for the more concentrated sample that was seeded with 5 × 10^9^ EVs, the same amount used in our previous experiment. Moreover, non- significant differences in the efficiency of uptake were observed when half of the EVs were seeded in the transwell, indicating that the number of EVs used in our experiments was in excess for this process (n = 3, P > 0.05). Furthermore, we only detected a small number of particles of the analyzed size in the lower chamber when no *B. subtilis* EVs were seeded (Supplementary Fig. S4), supporting that the number of particles released by Caco-2 cells did not interfere with our results. Overall, considering the data of both experiments measured by NTA, the calculated efficiency of uptake of *B. subtilis* EVs after 240 min of incubation ranged from 19% to 46%. This is also in agreement with the 29% obtained from fluorescence measurements.

To additionally verify the presence of intact transcytosed EVs, aliquots collected from the lower chamber were examined by Epi-fluorescence Microscopy. We observed intact EVs with a spherical shape in the aliquots collected from the lower chamber at 120 min (Fig. 4c). At that time, EVs showed an identical spherical shape compared to the EVs analyzed from aliquots collected from the upper chamber (data not shown) and from previously purified EVs^51^.

In our assays, the TEER value was constant during 240 min of EVs incubation (1730 Ω*cm^2^ ± 84 Ω*cm^2^). Therefore, it can be concluded that EVs transport across the Caco-2 cells exerted no effects on integrity and permeability of cell monolayers. Cell-free supernatant of the probiotic *Bifidobacterium lactis* increases tight junction integrity, while that from the pathogenic bacteria *Escherichia coli* O157:H7 (EHEC) induces damage on tight junction integrity. Furthermore, pre-administered *B. lactis* cell-free supernatant protects the tight junctions from EHEC-induced damage^63^. In this same work, the authors suggested the beneficial effects of specific probiotic bacteria in protecting intestinal epithelial cells from the deleterious effects of pathogenic bacteria was mediated by bioactive metabolites present in the culture supernatant from *B. lactis*. The authors concluded that further studies would be required to identify the active compounds and structural components of the supernatant that make possible the probiotic effect. Undoubtedly, it would be of great interest to elucidate whether EVs play a role in exerting directly these probiotic beneficial effects or are acting as delivery systems of bioactive compounds.

The concept of prokaryotic EVs transcytosis across intestinal epithelial cell has been previously proposed in the scientific literature but not demonstrated. Zhang *et al*. (2013) showed, in an *in vitro* model of intestinal porcine enterocytes, that EVs of *Helicobacter suis* were internalized, and the activity of cargo enzymes was detected in the lower chamber in a transwell system^64^. Kim *et al*. (2016) proposed a possible mechanism of action, in which *B. longum* EVs penetrate through intestinal epithelial cells, induce selective apoptosis of mast cells, and decreased allergy symptoms in a murine model of food allergy^45^. Furthermore, Behzadi *et al*. (2017) proposed that *L. rhamnosus* EVs could be lead up to the liver via the portal vein to induce their effects on hepatic cells^56^. On the other hand, *B. anthracis* produces toxin-laden EVs allowing a concentrated delivery of toxin components to target host cells, a mechanism that may increase toxin potency. These EVs are toxic for macrophages, and are capable of inducing a protective response in immunized mice^65^. In line with this, it would be interesting to study whether EVs from pathogens such as *B. anthracis* and *B. cereus* cross human intestinal epithelial cells, and check whether this step is an essential part of their virulent life cycle. If it is the case, the transcytosis of EVs across the epithelial cells of the GIT might be a universal mechanism shared by probiotic and pathogenic bacteria^64^.

### EVs internalization and movement in Caco-2 cells determined by confocal time lapse imaging

**Figure 5:**
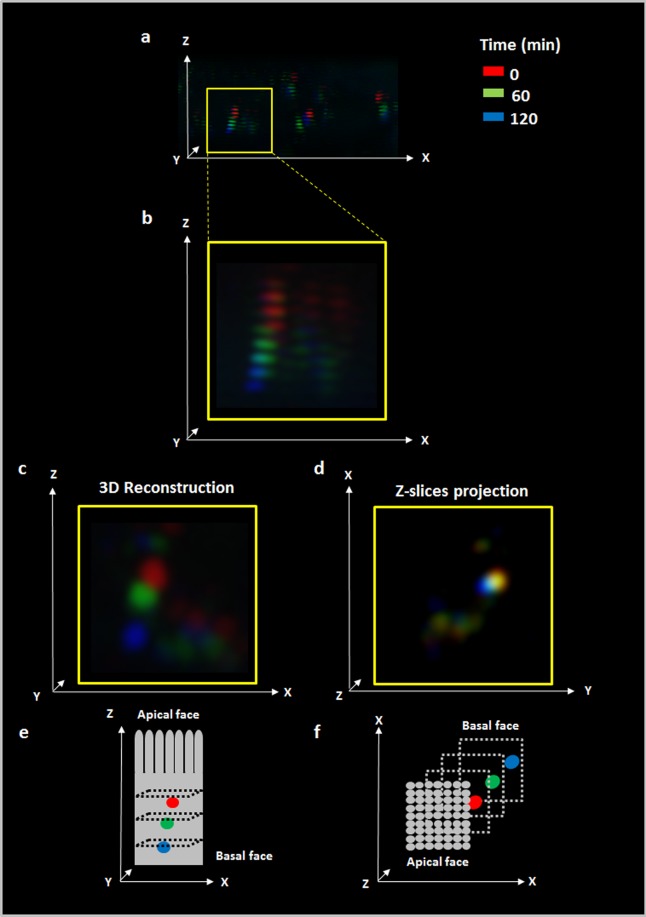
*B. subtilis* 168 EVs move through Z axis in Caco-2 cell monolayers. Caco-2 cell differentiated monolayers were incubated with CFSE-labeled EVs and series of time-lapse scans were taken at intervals of 10 min during 120 min. One color was assigned to three selected times 0 min (red), 60 min (green) and 120 min (blue) and then images were overlapped to infer EVs movement (FIJI software). (**a**) Panoramic view of a representative image. (**b**) Zoom of the selected area. (**c**) 3D Reconstruction of EVs within selected area. (**d**) Z-slices projection of the selected area. (**e**,**f**) are reference for time and spatial coordinates (XYZ) of **c** and **d** respectively.

In order to monitor the movement of EVs through Caco-2 cell monolayers, we carried out internalization assays and measured the transit of EVs by Confocal Time Lapse Imaging. An imaging area (XY) was fixed and consecutives slices obtained from Z-stacks were collected using 1.4 µm intervals. Series of time-lapse scans were taken at intervals of 10 min during the 120 min incubation with EVs. To infer EVs movement over time from these four coordinates (X, Y, Z and time) we assigned individual colours to each time point with an image processor (FIJI software). Then images were merged to observe the sequence of colours (time) through the XYZ space; *i.e*. we expected to find in the highest Z-stacks (apical face of Caco-2 cells) colours assigned to shorter time points, and in the lower Z-stacks (basolateral face of the cell) colours assigned to longer incubation time points. Figure 5a shows a panoramic view of different EVs where the sequence of colours corresponds to red (0 min), green (60 min) and blue (120 min) through the Z axis. As a representative example, Fig. 5b shows a zoom area where the sequence red, green and blue, corresponding from highest to lowest values of the Z axis, can clearly be seen. When this sequence of images was analysed through a 3D reconstruction software tool, the typical spherical shape of EVs and their movement through Z axis could be elucidated (Fig. 5c). Such a typical spherical shape from the 3D reconstruction came from 2–3 Z-stacks. Supplementary Video S1 shows the images superposition of 0, 60 and 120 min (Huygens Professional software).

If we consider the number of Z-stacks and the time required by EVs to pass through them, it is possible to calculate an approximate velocity of EVs transportation inside the Caco-2 cells. To do this, the number of Z-stacks crossed by one EV in the first hour can be calculated as the number of Z-stacks between the first red Z-stack and the first green Z-stack. In this case, the average of Z-stacks crossed by eight EVs was 2.69 Z-stacks ± 0.41 Z-stacks during the first hour of incubation. If we take into account the distance between Z-stacks (1.4 µm), the height of the monolayer of Caco-2 cells through the images (9.6 μm), and assuming that this speed is constant through the cells, we can estimate the time required by EVs to pass through the Caco-2 monolayers. This estimated time was 152 ± 28 min (n = 8), which is in accordance with our previous fluorescence results (Figs. 2 and 4). Supplementary Video S2 shows all Z-stacks of CFSE-EVs (green) internalized into the Caco-2 cells after 60 min of incubation (FIJI software).

On the other hand, when this image was analysed with a Z-slices projection, the merge of the 3 colours was obtained. This would indicate scarce movements on the XY plane (Fig. 5d). To measure the movement in X and Y axis, the coordinates in X and Y axis at 0, 60 and 120 min for 9 EVs were obtained using FIJI software (Supplementary Fig S5a). To quantify the movement, the mean value for the difference between coordinates at 0–60 and 60–120 min was calculated for these 9 EVs (Supplementary Fig. S5b). No statistically significant differences were found between movement on X and Y axis for the examined time periods (n = 9, p > 0.05) (Supplementary Fig. S5b). Based on these results, the movement on the X axis (1.5 μm ± 0.5 μm) and Y axis (1 μm ± 0.2 μm) resulted 70% lower compared to the travelled distance calculated on Z axis during the first hour (3.8 μm ± 0.6 μm). This last value was calculated as mentioned above considering the average of Z-stacks crossed by EVs in the first hour and the distance between Z-stacks.

There is an increasing amount of evidence showing that bacterial EVs can enter into eukaryotic cells and deliver their cargo^43,55^. Cells appear to take up EVs by a variety of endocytic pathways, including clathrin-dependent endocytosis, and clathrin-independent pathways such as caveolae-mediated uptake, macropinocytosis, phagocytosis, lipid raft-mediated internalization and direct membrane fusion^55^. Interestingly, pathogenic Gram-positive bacteria EVs seem to be internalized by eukaryotic cells through endocytic mechanism in several epithelial and macrophage cell lines^50,66,67^. Moreover, EVs could contact the host cells *via* cell membrane receptors to further initiate a cascade of intracellular signaling paths triggering the inflammatory response^66,68^. Thay *et al*. (2013) reported that the fusion of *S. aureus* EVs with the plasma membrane represents a route for delivery of a key virulence factor to human cells^69^. Except for membrane fusion, where the EVs content is released into the cytoplasm directly, whole EVs can enter into the recipient cell, whereby cargo release is mediated by fusion of the EVs membrane with the endosome membrane as described by Tian *et al*. (2013)^70,71^. Recently, a transport pathway for molecules from blood to brain cells was described by Villaseñor *et al*. (2017), involving dynamic intracellular tubules^72^. This transcytosis process regulated by sorting tubules is an additional pathway for receptor-mediated transcytosis that is distinct from non-selective transport like the caveolae class and trans-endothelial channels.

Our results suggest that Caco-2 cells were able to take up intact EVs from *B. subtilis* and, at least part of them, are not fused with cell membranes and are transported and secreted at the opposite cell surface side. Possibly, EVs populations, heterogeneous in terms of content, could enter into the cell by more than one way. Further studies should elucidate how bacterial EVs are transported across the intestinal epithelial cell barrier.

## Conclusions

In this work, we demonstrated that intact EVs from *B. subtilis* could be transported across Caco-2 cells differentiated to enterocyte-like cells. Images from CLSM demonstrated that *B. subtilis* EVs uptake and internalization by Caco-2 cells were time-dependent. Using a combination of Fluorescence and NTA measurements in a transwell system, we were able to show that intact EVs could be translocated from the upper to the lower chamber with an average efficiency of uptake of 30% after a 240 min incubation period and this process was also dose-dependent. Using a Time Lapse Microscopy technique we elucidated that EVs movement was mainly through Z axis and slightly on X and Y axis. In conclusion, our studies could partially explain one of the possible mechanisms of communication between probiotics and host. This mechanism could be a first step that allows EVs to reach the bloodstream and consequent delivery to extraintestinal tissues and organs. The encapsulation of proteins, siRNA, mRNA, and chemotherapeutics into EVs could have practical implications in food, nutraceuticals and clinical therapies.

## Methods

### Materials

*Bacillus subtilis* 168 was obtained from the *Bacillus* Genetic Stock Center. Brain Heart Infusion (BHI) was purchased from Merck Millipore. Pore-size membranes: 0.8, 0.65 and 0.45 µm were obtained from GE Osmonics, and 100 kDa cut-off filter from Sartorius. Caco-2 cell line was a kind gift of Dr. Guillermo H. Docena (Instituto de Estudios Inmunológicos y Fisiopatológicos, La Plata, Argentina). Dulbecco’s Modified Eagle’s Medium (DMEM), fetal bovine serum (FBS), TrypLE, penicillin and streptomycin were purchased from Gibco. Rhodamine Phalloidin and To-Pro3 Iodide were purchased from Thermo Fisher Scientific, and 5-(6) carboxyfluoresceindiacetate N-succinimidyl ester (CFSE) from Invitrogen. Poly-D-Lysine, transwell polycarbonate membranes, 24 and 96 well plates were obtained from Corning.

### Bacterial culture and extracellular vesicles isolation

100 ml of BHI medium was used for *Bacillus subtilis* 168 growth under the following conditions: 37 °C, 200 rpm of agitation during 18 h. EVs were isolated according to the protocol previously detailed in Domínguez Rubio *et al*.^51^. Briefly, in order to remove *B. subtilis* cells, cultures were centrifuged for 25 min at 4000 g, at 25 °C. Supernatant was filtered through 0.8, 0.65 and 0.45 μm to remove cellular debris. Centricon ultrafiltration system (cut off = 100 kDa) was used for concentration the filtrated supernatant. Then, the concentrated supernatant was filtered again through 0.45 μm filter to remove the aggregated material. This filtered supernatant was centrifuged 120 min at 110,000 g, 4 °C using a SW 41 Ti rotor (Beckman Optima L-80). The resulting pellet was washed with a phosphate buffered saline solution (PBS). EVs contained in the pellet were labeled, resuspended in PBS or Dulbecco’s modified Eagle’s medium (DMEM), filtrated by a 0.45 mm filter and used in further determinations.

### Nanoparticle tracking analysis (NTA)

Particle number and size distribution was obtained using a Nanosight NS3000 System equipped with a 488 nm laser. To this end, EVs resuspended in PBS were injected in the device. Three videos of 10 seconds were obtained at camera level 9, at 25 °C for each sample. NTA 3.2 software was used for data analysis with a detection threshold 3 and maintaining other settings at default. PBS was completely particle-free at these settings.

### EVs labeling with 5-(6) carboxyfluorescein diacetate N-succinimidyl ester (CFSE)

EVs were stained with CFSE at 10 mM final concentration in filtered PBS for 30 min at 37 °C and washed twice with filtered PBS^51,73^. This non-fluorescent dye diffuses across EVs cell membrane where esterases of the plasmatic membrane cleave its acetate group forming fluorescent CFSE. This fluorogenic dye is now not permeable and, consequently, is confined into EVs.

### Caco-2 cell culture and differentiation

Caco-2 cells (passage number 50–60) were growth in Dulbecco’s Modified Eagle Medium (DMEM), containing glucose, 10% heat-inactivated fetal bovine serum (FBS), 50 U/ml penicillin and 50 μg/ml streptomycin. Caco-2 cells were incubated at 37 °C and humidified atmosphere with CO2 5% and media was renewed at 48 h. Cells were routinely subcultured by tripsinization (TrypLE 2X diluted in PBS 1 mM EDTA) when they reached 80% confluence. For uptake and internalization assays by Confocal Laser Scanning Microscopy, cells were seeded on 12 mm glass coverslips coated with Poly-D-Lysine (100 µg/ml) 1 h at 37 °C in 24 well plates at a seeding density 2.5 × 10^5^ cells/well. For Confocal Time Lapse Microscopy experiments, cells were seeded on live-cell imaging chambers at density of 2.5 × 10^5^ cells/well. For transcytosis assays, cells were seed on transwell polycarbonate membranes of 0.4 μm pore size and 0.8 cm^2^ filter area at a seeding density of 7 × 10^5^ cells/transwell. For MTT assay cells were seeded on 96 well plates at a seeding density of 1 × 10^4^ cells/well. Cultured cells were maintained for 14 days until obtaining a mature differentiated monolayer.

### EVs uptake and internalization in Caco-2 cells by Confocal Laser Scanning Microscopy (CLSM)

Differentiated Caco-2 monolayers were obtained as specified above, washed with PBS and incubated with 1.3 × 10^9^ labeled EVs resuspended in basal DMEM at different times (0, 5, 15, 30 45, 60, 120, and 240 min) at 37 °C in a humidified atmosphere containing 5% CO_2_^64^. After incubation, Caco-2 cells were fixed with paraformaldehyde 4% (w/v) in PBS for 20 min at 25 °C. After several washes with PBS, cells were permeabilized for 10 min with Triton X-100 dissolved in PBS (0.05%, v/v) at 25 °C. Following washes, non-specific binding sites were blocked with 3% (w/v) BSA in PBS 1 h at RT. After that, cells were incubated with Rhodamine Phalloidin 0.1% (w/v) and To-Pro3 Iodide 0.1% (w/v), both in PBS 1 h at RT. Finally, coverslips were mounted with Mowiol and examined by CLSM (Olympus FV 1000 module). CFSE dye has a λexc = 494 nm and λem = 521 nm, which was measured using 488 nm laser excitation and 505/525 band pass filter for detection^73^. Rhodamine Phalloidin has a λexc = 540 nm and λem = 565 nm, which is measured using 543 nm laser excitation and 560/620 band pass filter for detection^74^. To-Pro3 Iodide has a λexc = 642 nm and λem = 661 nm, which is measured using 633 nm laser excitation and 655/755 band pass filter for detection^75^. Z-stacks were collected using 1.4 µm intervals. Images (1024 × 1024 pixels) showing the fluorescence were taken using a PLAPON 60X water objective N.A. = 1.42 (1 μm = 19.3 pixel). Digital pictures were processed with FIJI software (National Institutes of Health, Baltimore, USA). 200 cells per sample were analyzed to quantify EVs and CFSE fluorescence. The experiments were performed in triplicate.

### Monolayer integrity for transcytosis assays

Caco-2 cells were differentiated on transwell polycarbonate membranes. Measurements of the trans-epithelial electrical resistance (TEER) were performed to determine the integrity of and differentiation of Caco-2 cells monolayers^20^. TEER was measured through the monolayer formation every 2–3 days. Measurements of Ω (electric resistance) were performed by an Epithelial Voltohmmeter EVOM2 with sterilized electrodes. TEER was calculated as:

$${\rm{TEER}}=[\Omega \,({\rm{cell}}\,{\rm{inserts}})-\Omega \,({\rm{cell}}\, \mbox{-} \,{\rm{free}}\,{\rm{inserts}})]\times {\rm{filter}}\,{\rm{area}}\,{({\rm{cm}}}^{2})$$

Monolayers with stabilized TEER on day 14 after Caco-2 cells seeding were used for transcytosis assays.

### Transcytosis assays

The upper and lower chambers of the transwell were washed with PBS to eliminate all residual culture medium and the lower chamber was replaced with 200 µl of basal DMEM. CFSE-labeled EVs were gently mixed in basal DMEM and approximately 5 × 10^9^ EVs resuspended in 150 μl were incubated with Caco-2 in the upper chamber of the transwell. Fluorescence was measured using a fluorescence microplate reader POLARstar Omega and the number of EVs was quantified by NTA. To this end, 190 μl of medium were collected from the lower chamber at different times of incubation (0, 15, 30 45, 60, 120, and 240 min), and fresh medium was added to maintain the lower chamber volume. On the other hand, 10 μl aliquots of the medium were collected from the lower chamber and were examined by fluorescence microscopy. Images were taken using an Olympus IX71 inverted Epi-fluorescence microscope with an Oil 60X/NA 1.42 UPLAN SAPO objective. Images were captured with a Hamamatsu Photonics ORCA-ER camera. Digital pictures were processed with FIJI (National Institutes of Health, USA).

To calculate uptake efficiency the volume from the upper chamber (150 μl) was diluted to 190 μl (volume from lower chamber) to measure fluorescence and calculate as:

$${\rm{Uptake}}\,{\rm{efficiency}}=({\rm{fluorescence}}\,{\rm{lower}}\,{\rm{chamber}}/{\rm{fluorescence}}\,{\rm{upper}}\,{\rm{chamber}})\times 100$$

Transwells without cells were used as control of passive diffusion^76^. TEER was measured during the incubation with EVs to verify the barrier integrity of the differentiated Caco-2 cell monolayer^77^. The experiment was performed in triplicate.

### Confocal time lapse imaging

Labeled EVs were resuspended in basal DMEM. Caco-2 monolayers were obtained as specified above, washed with PBS and incubated with 115 µg of labeled EVs at 37 °C in a humidified atmosphere containing 5% CO_2_ (live chamber controller)^78^. An imaging area (XY) was fixed and 20 slices and Z-stacks were collected using 1.4 µm intervals. Series of time-lapse scans were taken at intervals of 10 min during 120 min. The fluorescent images were taken using an Olympus FV 1000 module using the same microscopy settings described above. Digital pictures were processed with FIJI software (National Institutes of Health, Baltimore, USA) and Huygens Professional software (Scientific Volume Imaging, Netherlands).

### MTT assay

This colorimetric assay measures the reduction of yellow 3-(4,5-dimethylthiazol-2-yl)−2,5- diphenyltetrazolium bromide (MTT) by mitochondrial succinate to purple colored formazan crystals. Since reduction of MTT can only occur in metabolically active cells the level of activity is a measure of cellular proliferation, viability and cytotoxicity^79^. Briefly, cells were grown in 96 well plates and incubated with approximately 5 × 10^9^ EVs suspended in basal DMEM at 37 °C for 0, 5, 15, 30 45, 60, 120, and 240 min. Then, cells were incubated with 200 µl MTT solution (5 mg/ml MTT in basal DMEM) for 120 min at 37 °C. The medium was removed and the crystals formed were dissolved in 200 µl of DMSO per well. Absorbance was measured at 570 nm and 690 nm (background) using POLARstar Omega microplate reader. The relative cell viability was calculated as Abs570nm-Abs 690 nm. The assay was performed in octuplicate.

### Statistical analysis

The number of internalized EVs and fluorescence units per cell analyzed by CLSM, the data obtained from MTT assay and the number of EVs measured by NTA were analyzed by means of a one-way ANOVA in a completely randomized design. The data obtained from EVs movement on X and Y axis analyzed by Confocal Time Lapse Imaging were analyzed by means of a 2-way ANOVA with interactions (axis and time) in a completely randomized design. Tukey’s test was used for post hoc comparisons. The assumptions of normality and homoscedasticity were studied analytically by the Shapiro–Wilks and the Levene tests, respectively. Results were considered significant at p < 0.05. Statistical analyses were performed using the statistical program Infostat (Universidad Nacional de Córdoba, Argentina).

## Supplementary information

Supplementary Info.

Supplementary Video 1.

Supplementary Video 2.
