# Supplementary material for: Transcytosis of Bacillus subtilis extracellular vesicles through an in vitro intestinal epithelial cell model
Source: Sci Rep. 2020 Feb 20;10:3120. doi: 10.1038/s41598-020-60077-4 (PMC7033168; doi:10.1038/s41598-020-60077-4)
Supplement: Supplementary file 1 — Supplementary Info. [file 41598_2020_60077_MOESM1_ESM.pdf]

Supplementary Info:

Transcytosis of *Bacillus subtilis* extracellular vesicles through an *in vitro* intestinal epithelial cell model

Ana Paula Domínguez Rubio[1,2,ǂ],Jimena Martínez[1,2,ǂ],Marcos Palavecino[1,2], Federico Fuentes[3], Christian Miquel Sánchez-López[4,5], Antonio Marcilla[4,5], Oscar Edgardo Pérez[1,2,*]and Mariana Piuri[1,2,*]

[1] Universidad de Buenos Aires, Buenos Aires, Argentina.
[2] Instituto de Química Biológica de la Facultad de Ciencias Exactas y Naturales, Universidad de Buenos Aires, Consejo Nacional de Investigaciones Científicas y Técnicas, Buenos Aires, Argentina.
[3] Instituto de Medicina Experimental, Consejo Nacional de Investigaciones Científicas y Técnicas, Buenos Aires, Argentina.
[4] Àrea de Parasitologia, Departament de Farmàcia i TecnologiaFarmacèutica i Parasitologia, Universitat de València, Burjassot, Valencia, Spain;
[5] Joint Research Unit on Endocrinology, Nutrition and Clinical Dietetics, Health Research Institute La Fe, Universitat de Valencia, Valencia, Spain.
[*]Correspondence:oscarperez@qb.fcen.uba.ar or mpiuri@qb.fcen.uba.ar
[ǂ] These authors contributed equally to this work.

***Contact information:**

Mariana Piuri
mpiuri@qb.fcen.uba.ar

Oscar E. Pérez
oscarperez@qb.fcen.uba.ar

Supplementary Video 1. *B. subtilis* 168 EVs move through Z axis in Caco-2 cell monolayers. Caco-2 cell differentiated monolayers were incubated with CFSE-labeled EVs and series of time-lapse scans were taken at intervals of 10 min during 120 min. For EV trajectory record inside the cell, images from three selected times (0, 60 and 120 min) are overlapped and visualized through the rotation on the Z axis (Huygens Professional software).

Supplementary Video 2. *B. subtilis* 168 EVs internalized by Caco-2 cell monolayers. Caco-2 cell differentiated monolayers were incubated with CFSE-labeled EVs and series of time-lapse scans were taken at intervals of 10 min during 120 min. CFSE-labeled EVs were internalized by Caco-2 cells. Images from all Z-stacks at 60 min of incubation are visualized through the rotation on the Y axis (FIJI software).

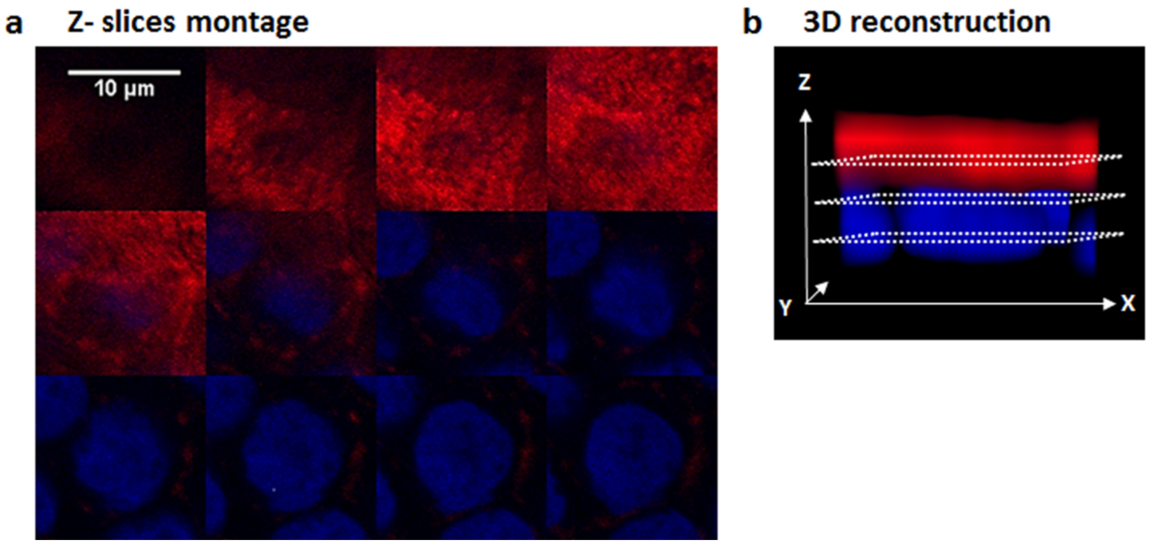

Supplementary Figure S1. Caco-2 cells after attaining confluence were differentiated and presented enterocyte-like cells morphology, i.e. presence of well-developed apical microvilli (brush border, in red) and basal nuclei (in blue). Nuclear DNA was stained with To-Pro3 and actin filaments were stained with Rhodamine Phalloidin and visualized by CLSM. Digital pictures were processed with FIJI software. (**a**) Z-slices montage. (**b**) 3D reconstruction.

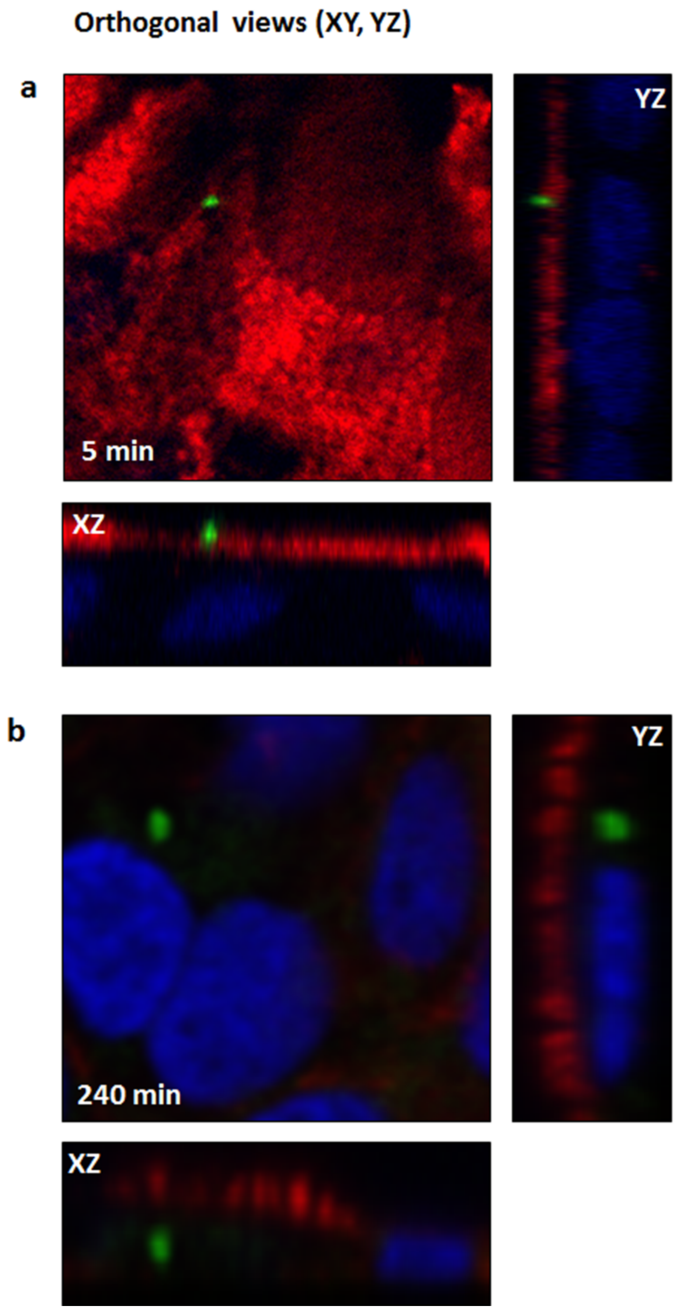

Supplementary Figure S2. ***B. subtilis*** **EVs were taken up by Caco-2 cells.** Caco-2 cell differentiated monolayers were incubated with CFSE-labeled EVs for different times. After incubation cells nuclear DNA was stained with To-Pro3 and actin filaments were stained with Rhodamine Phalloidin. Images were obtained by CLSM. Digital pictures were processed with FIJI software. Orthogonal views (XY, YZ) of representative images of (**a**) 5 min and (**b**) 240 min are offered.

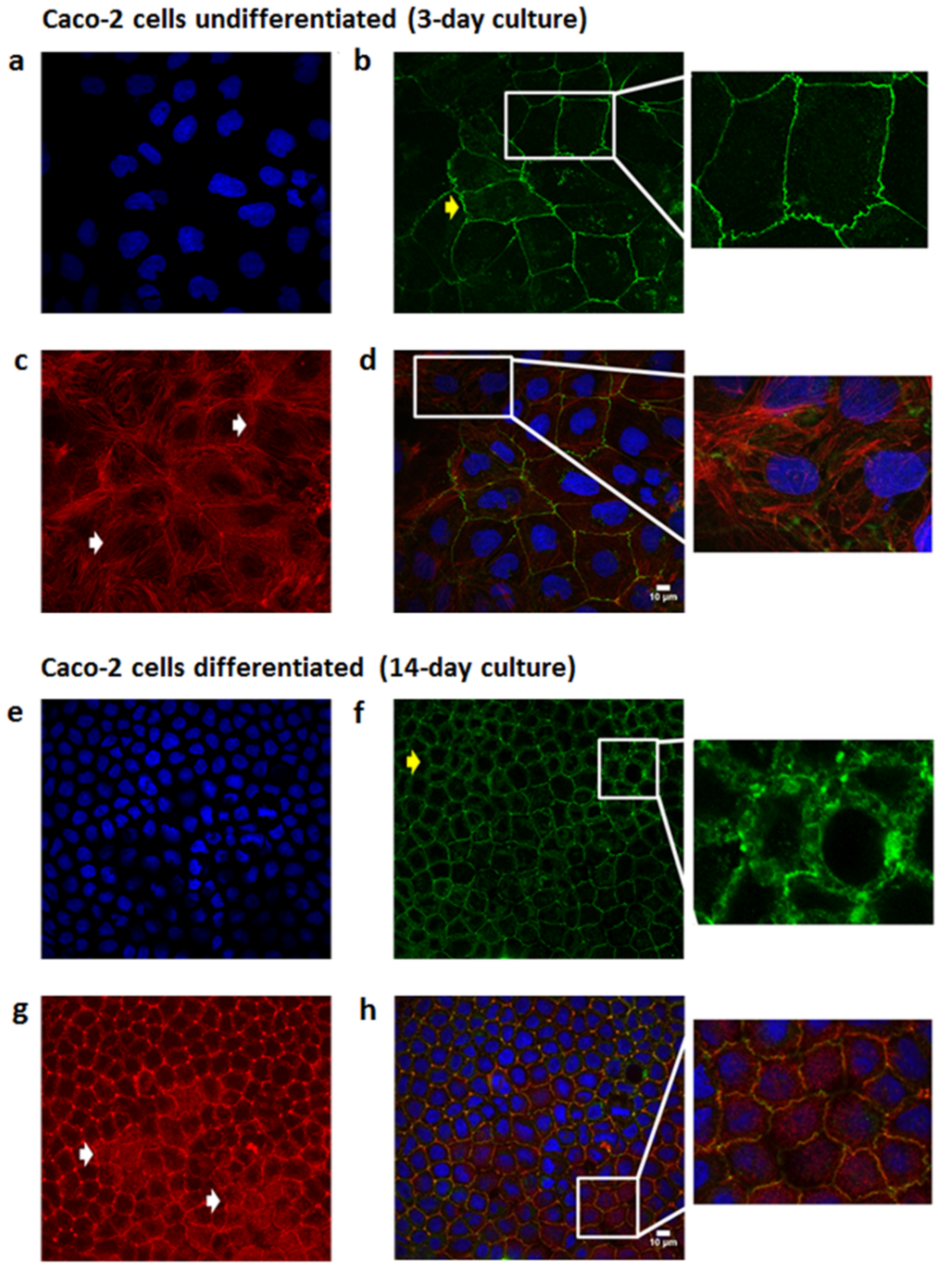

Supplementary Figure S3. Comparative analysis of cell differentiation by occludin immunofluorescence. Caco-2 cells after 3 days (**a**-**d**), and 14 days (**e**-**h**), of culture were stained withTo-Pro3 (**a** and **e**), anti-occludin primary antibodies and Alexa 488 labeled secondary antibodies (**b** and **f**), and Rhodamine Phalloid (**c** and **g**). Merge images (**d** and **h**). The yellow arrow in **b** shows occluding forming a thin uncontinuous staining at the cell periphery, and the white arrows in **c** indicate the actin cytoskeleton through all the cytoplasm, both characteristic of an undifferentiated monolayer. The yellow arrows in **f** indicate a stronger continuous signal of occluding at the cell periphery, and the white arrows in **g** show apical actin, both characteristic of differentiated monolayer.

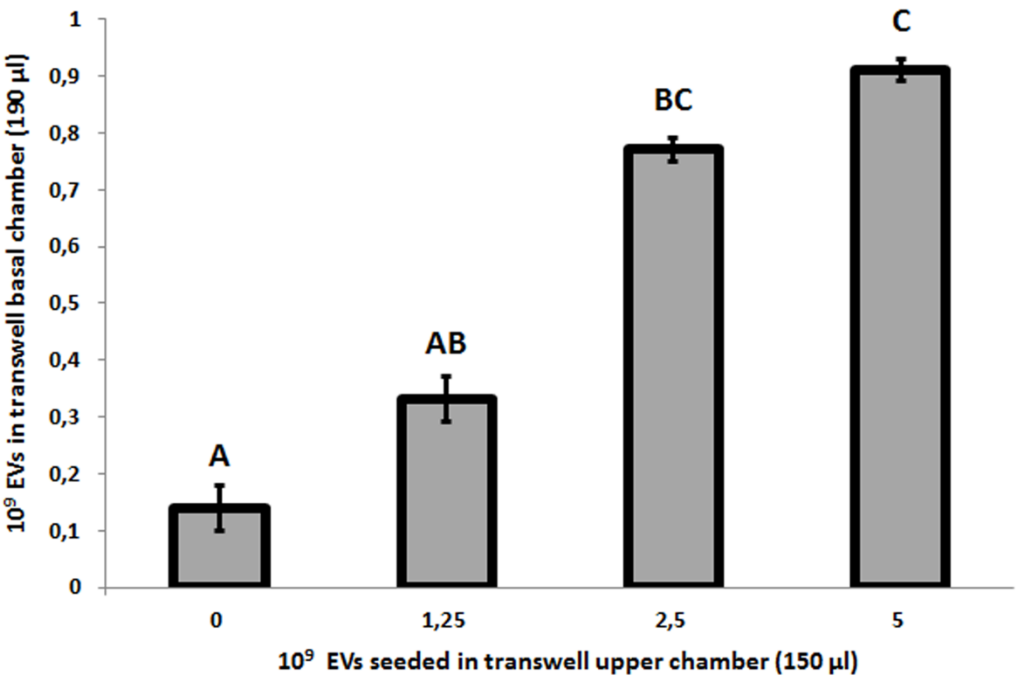

Supplementary Figure S4. *B. subtilis* EVs are transported across Caco-2 cells in a dose-dependent manner. *B. subtilis* EVs were incubated with Caco-2 cell differentiated monolayers in the upper chamber of a transwell system during 240 min of incubation. The number of EVs of the medium collected from the lower chamber was measured by NTA. Mean ± SD. One-way ANOVA with Tukey's post hoc test were carried out (n=3). Means with a common letter are not significantly different ($p>0.05$).

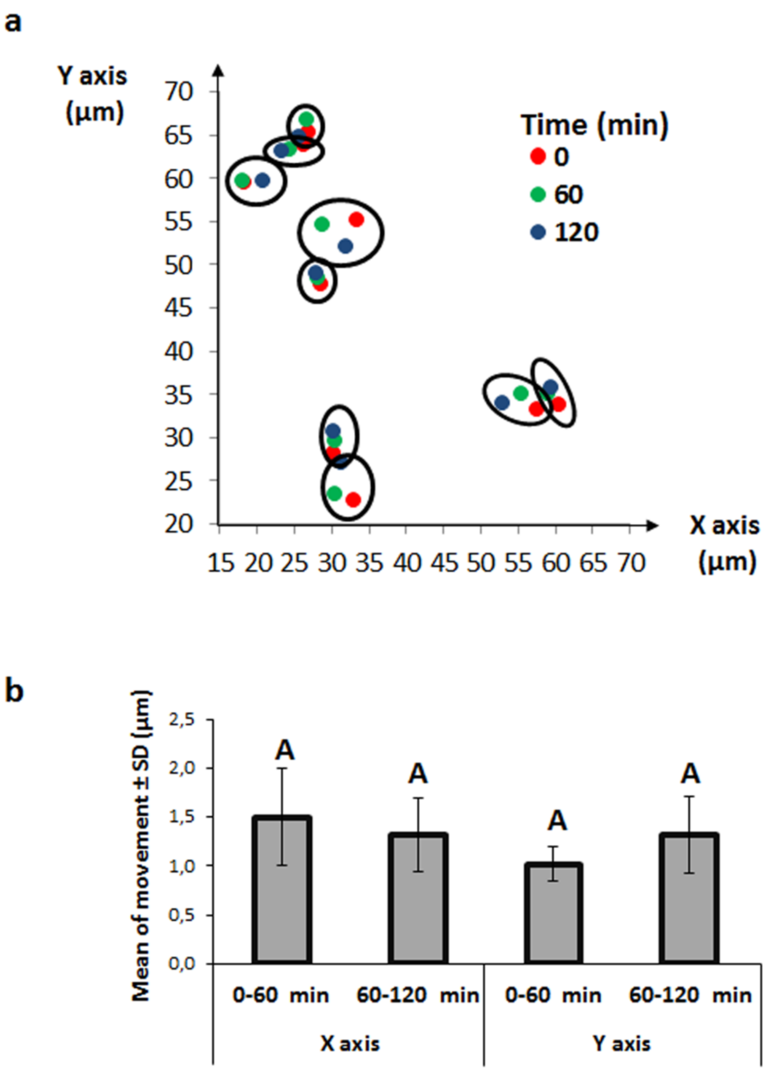

Supplementary Figure S5. *B. subtilis* 168 EVs movement on X and Y axis in Caco-2 cell monolayers. Caco-2 cell differentiated monolayers were incubated with CFSE-labeled EVs and series of time-lapse scans were taken at intervals of 10 min during 120 min. (**a**) Schematic representation of spatial coordinates (XY) of 9 EVs at three selected times 0, 60 and 120 min. Each black circle enclosed one EV at 0 min (red), 60 min (green) and 120 min (blue). (**b**) Mean ± SD for the difference between coordinates at 0-60 and 60-120 min on X and Y axis. 2-way ANOVA with interactions (axis and time) (n=9). Means with a common letter are not significantly different ($p>0.05$).
